# NuBBE_DB_: an updated database to uncover chemical and biological information from Brazilian biodiversity

**DOI:** 10.1038/s41598-017-07451-x

**Published:** 2017-08-03

**Authors:** Alan C. Pilon, Marilia Valli, Alessandra C. Dametto, Meri Emili F. Pinto, Rafael T. Freire, Ian Castro-Gamboa, Adriano D. Andricopulo, Vanderlan S. Bolzani

**Affiliations:** 1 Nuclei of Bioassays, Biosynthesis and Ecophysiology of Natural Products (NuBBE), Department of Organic Chemistry, Institute of Chemistry, Sao Paulo State University - UNESP, 14800-060 Araraquara, SP Brazil; 2 Centro de Imagens e Espectroscopia in vivo por Ressonância Magnética, Institute of Physics of Sao Carlos, University of Sao Paulo - USP, 13566-590 Sao Carlos, SP Brazil; 3 Laboratório de Química Medicinal e Computacional (LQMC), Centro de Pesquisa e Inovação em Biodiversidade eFármacos, Institute of Physics of Sao Carlos, University of Sao Paulo - USP, 13563-120 Sao Carlos, SP Brazil

## Abstract

The intrinsic value of biodiversity extends beyond species diversity, genetic heritage, ecosystem variability and ecological services, such as climate regulation, water quality, nutrient cycling and the provision of reproductive habitats it is also an inexhaustible source of molecules and products beneficial to human well-being. To uncover the chemistry of Brazilian natural products, the Nuclei of Bioassays, Ecophysiology and Biosynthesis of Natural Products Database (NuBBE_DB_) was created as the first natural product library from Brazilian biodiversity. Since its launch in 2013, the NuBBE_DB_ has proven to be an important resource for new drug design and dereplication studies. Consequently, continuous efforts have been made to expand its contents and include a greater diversity of natural sources to establish it as a comprehensive compendium of available biogeochemical information about Brazilian biodiversity. The content in the NuBBE_DB_ is freely accessible online (https://nubbe.iq.unesp.br/portal/nubbedb.html) and provides validated multidisciplinary information, chemical descriptors, species sources, geographic locations, spectroscopic data (NMR) and pharmacological properties. Herein, we report the latest advancements concerning the interface, content and functionality of the NuBBE_DB_. We also present a preliminary study on the current profile of the compounds present in Brazilian territory.

## Introduction

Historically, natural products have been recognized as the primary source of compounds for medicines, cosmetics and food. Today, natural products continue to be an important resource for technological and socioeconomic development and for maintaining human well-being^1–4^.

As a megadiverse country, Brazil accounts for 10–20% of known living species in the world. However, a major part of the biological and chemical biodiversity in Brazil remains unexplored^2, 4, 5^. The last two centuries have been characterized by extensive degradation of biodiversity due to disorganized economic growth based on agribusiness (soy and sugarcane monocultures and extensive livestock), urbanization and logging exploration. These practices not only disrupt the balance of the ecosystem but also threaten the entire genetic heritage of flora and fauna^2, 6, 7^. The latest review in the Red Book of Brazilian Flora noted that, in the Atlantic Forest and Cerrado ecoregions, the Asteraceae, Bromeliaceae and Orchidaceae families are critically threatened, primarily because of anthropic effects^8^. Consequently, an increase in the effects associated with climatic and ecological changes has been observed in addition to the loss of an arsenal of high-value metabolites^5, 6, 9, 10^.

One approach to mitigate the progressive loss of biodiversity is the preparation of diagnostic reports evaluating the existing biological, chemical, and climatic states of habitats and ecoregions^2, 10, 11^. The Intergovernmental Science-Policy Platform on Biodiversity and Ecosystem Services (IPBES), organized by UNESCO, has acted as a mediator between the scientific community and policymaking institutions by preparing responses and solutions for global biodiversity problems. Part of the strategy of the IPBES involves the aggregation of peer-reviewed works on a range of scientific topics in databases or multidisciplinary platforms^11^.

In this sense, “big data” libraries have played a crucial role as centers for the acquisition, organization and distribution of knowledge intended for the resolution of issues related to the most important human and environmental topics. Taxonomy and ecology repositories such as Species 2000^12^, Catalogue of Life^12^ and the Global Biodiversity Information Facility^13^ (GBIF) have offered cataloguing services and addressed issues involving species distribution and occurrence in unique ecological niches. Furthermore, databases have been crucial in the development of multidisciplinary research fields such as medicinal chemistry, chemosystematics, ethnopharmacology and “omics” approaches. For example, many genomic studies, such as the mapping of the human genome, have used GenBank^14^ and the DNA Databank of Japan^15^ (DDBJ); many pharmacological, computational and proteomic studies have employed the Protein Data Bank^16^ (PDB), the Human Proteome Map^17^ and the Peptide Atlas^18, 19^; and many metabolomic studies have relied on the Human Metabolome Database^20^ (HMDB), the Golm Metabolome Database^21^, Global Natural Product Social Molecular Networking (GNPS)^22^, Metlin^23, 24^, the Biological Magnetic Resonance Bank^25^ (BMRB) and MassBank^26^. Additionally, the properties contained in compound libraries, including Pubchem^27^, ChemSpider^28^, Zinc^29^, PK/DB^30^, BindingDB^31^, ChemBank^32^, ChEMBL^33^ and DrugBank^34^, have been used extensively in drug discovery projects.

Multidisciplinary platforms are fundamental to a deeper comprehension of systemic and multifactorial events, e.g., hierarchical evaluation between genotype and phenotype levels, as well as inferences on epigenetic variations and large-scale phenomena that characterize climatic changes and ecological perturbations^6, 7, 35, 36^. Databases should not only provide a series of biological, geographic, climatic or chemical data but also connect topics in a uniform and practical language, converting unrelated and scattered information into a knowledge repository. This is not a simple task, and it requires the collaboration of governmental agencies, academic communities and multiskilled researchers.

Under these circumstances, the Ministry of Science, Technology, Innovation and Communication (MCTIC) of Brazil, in collaboration with funding agencies such as the National Council for Scientific and Technological Development (CNPq) and São Paulo Research Foundation (FAPESP), has supported multidisciplinary programs that involve online platforms addressing the conservation and sustainable use of Brazilian biodiversity. The FAPESP Research Program on Biodiversity Characterization, Conservation, Restoration and Sustainable Use (BIOTA-FAPESP) is a successful example of how organized information about Brazilian biodiversity can contribute to the sustainable use of natural resources, support the development of social and economic models and assist in the drafting of environmental laws for the state of São Paulo^2^.

Under these conditions, the Nuclei of Bioassays, Ecophysiology and Biosynthesis of Natural Products Database (NuBBE_DB_) was established in 2013 as a library with 640 compounds. The NuBBE_DB_ was intended to provide molecular descriptors and chemical structures of the natural products studied in NuBBE laboratories for molecular modeling and medicinal chemistry studies^37^. The worldwide impact of this initially small chemical database, which has proved to be a valuable resource for new drug design and dereplication studies, has encouraged its expansion^38–41^. Since 2015, continuous efforts have been made to expand its content, include more diverse natural sources and establish a comprehensive compendium of available biogeochemical information about Brazilian biodiversity.

Herein, we describe the NuBBE_DB_ online web server (version 2017) and its available services, organizational aspects, contents, and tools. Since its creation in 2013, the NuBBE_DB_ has changed considerably in terms of content and computational layout, necessitating a report of updated information on these achievements and results. We also report for the first time the distribution of natural products within the Brazilian biomes, the relationship of this distribution with species occurrence and the association of biological activities with metabolic classes and species.

## Results

### Enhancements in the content, information and coverage of the NuBBE_DB_

#### Overview, literature search and content expansion

The NuBBE_DB_ is undergoing continuous development to become a larger and more complete database of Brazilian natural product chemistry. In collaboration with the CNPq, we searched the Lattes database for the keywords “natural products” and “Brazilian biodiversity” between 1950 and 2015. The Lattes database is a platform containing curriculum data from mainly Brazilian researchers. This search led to a list of more than thirty-two thousand scientific papers, which constitute the current source of information for the NuBBE_DB_. Although these papers are a great foundation, in the future, we need to address the gap in information regarding studies of Brazilian organisms performed by non-Brazilian researchers since these data were not available in our primary search.

In the last four years, the number of compounds in the NuBBE_DB_ has increased by 200%, and the database currently includes more than 2,000 natural products and derivatives. This information was extracted from more than 1,500 papers, which have already been analyzed following established inclusion criteria (see the *Criteria for paper inclusion* section for details). To date, we estimate that the NuBBE_DB_ contains approximately 5% of the information available in the more than 32,000 papers searched in collaboration with the CNPq.

#### Criteria for paper inclusion

**Figure 1:**
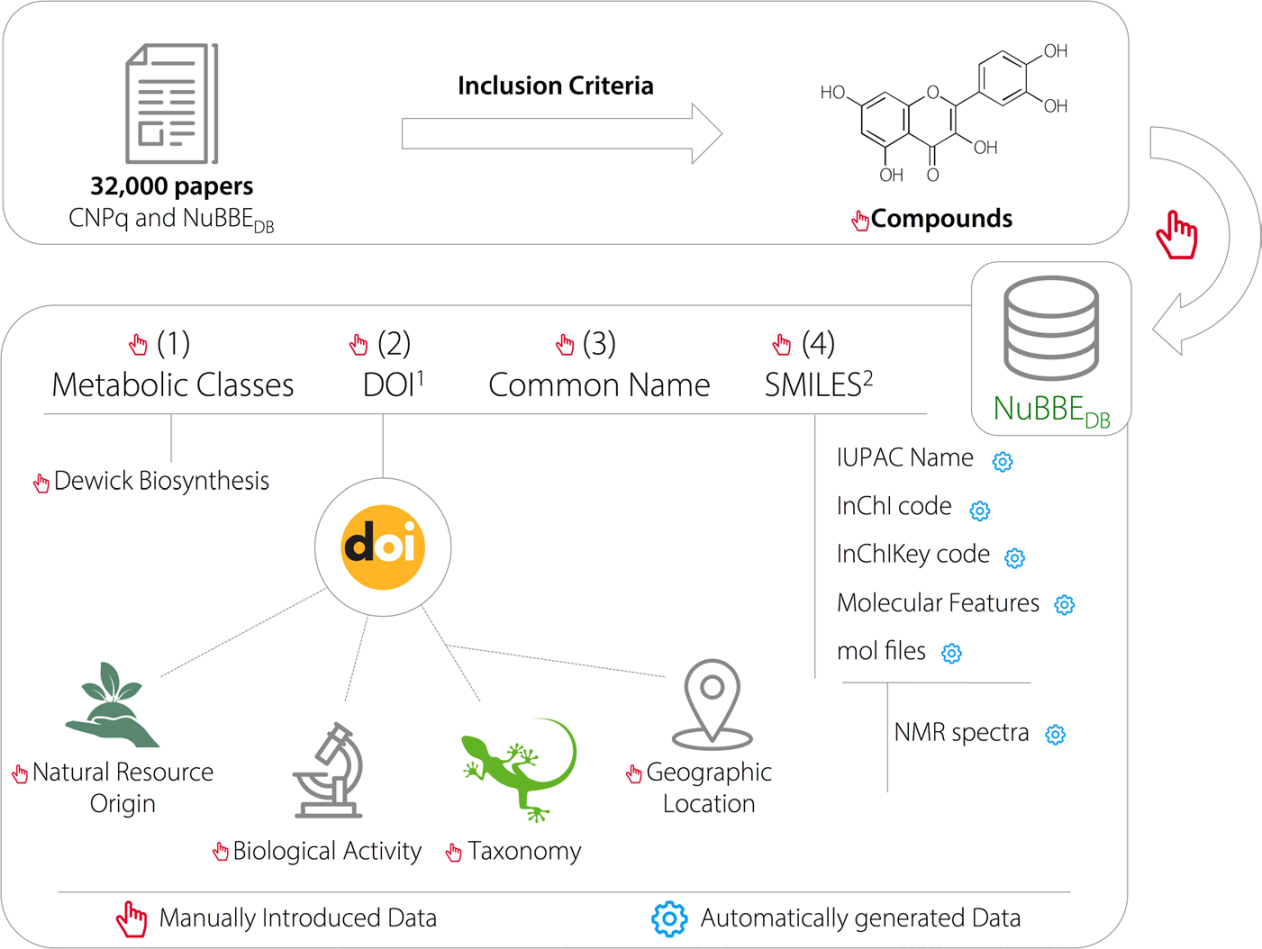
Schematic representation of the hierarchical process of data entry for the NuBBE_DB_ website. This workflow is divided into two steps. First, in collaboration with governmental databases, a literature search is performed to rationalize and organize all available information about Brazilian biodiversity. Subsequently, data are analyzed, and information such as structure, biological properties, source and geographical location is manually introduced into the database. The IUPAC name, InChI codes, molecular features, MOL files and NMR spectra are automatically generated from SMILES.

Each scientific paper is evaluated to extract information about natural and semi-synthetic compounds and biotransformation products. Total syntheses of natural products and their analogues are not yet included in this database. To guarantee data quality, the publications must comply with the following two criteria: (i) the scientific paper must contain a digital object identifier (DOI), and (ii) all studies must indicate that the identified, isolated or modified metabolites were obtained from organisms (plants, fungi, bacteria, marine organisms, etc.) found in Brazilian territory, as shown in Fig. 1.

#### Improvements in the database website structure

The integration of biogeographic aspects (mapping, distribution and occurrence of species) with biological and chemical information provides a robust knowledge base to supply information to several scientific communities and assist policy makers with preservation strategies for and sustainable use of biodiversity^2^. Since our latest release in 2013, the computational structure was enhanced to assume a hierarchical form, as summarized in Fig. 1. The first level includes the following four primary aspects: (1) metabolic class, consisting of a drop-down list of 14 classes and subclasses based on Dewick’s biosynthesis^42^; (2) DOI, used as a key feature to link to the second level of information, which is source, biological activity, species information and geographic location; (3) the common name assigned by a paper’s authors; and (4) SMILES (Simplified Molecular-Input Line-Entry System) notation, a linear notation describing a compound’s chemical structure, which is the most vital information in the database. Additionally, SMILES notations are the basis for the automatic generation of the third level of information, including the 2D image structure, IUPAC name, monoisotopic mass, molecular mass, molecular volume, numbers of hydrogen bond donors and acceptors, octanol/water partition coefficient (cLogP), number of rotatable bonds (nRotb), topological polar surface area (TPSA), number of violations of Lipinski’s rule of 5 (Ro5), 3D chemical structure (.MOL2 and .MOL format files), InChI and InChIKey codes and predicted ^1^H and ^13^C NMR spectra.

The NuBBE_DB_ team, in collaboration with ACD/Labs, has devoted effort to integrating NMR prediction software with the database, primarily for metabolomic studies. The main challenges associated with these studies are the identification and quantification of the overall metabolic profile for biological matrices^43–45^. Thus, tools and methodologies that can analyze all chemical diversity (stereochemical and structural diversity) and concentration ranges (from micrograms to grams) are crucial^44–47^. In this sense, standardized compound libraries are useful as interaction networks between structural data, taxonomic information and biological activities and they can lead to the faster identification of biomarkers detected in metabolomic studies. Predictions are made using reliable and robust algorithms and assuming that the solvent is “undefined”, i.e., it is regarded as a mixture of all solvents. For various purposes, we have simulated 600 MHz (hydrogen frequency) and 150 MHz (carbon frequency) spectra using 65,000 points and spectral windows set from 0 to 14 ppm for ^1^H NMR and from 0 to 220 ppm for ^13^C NMR. In addition, a peak-picking list, coupling constants and signal intensities are provided. This initiative is interesting because it will allow for evaluation of relationships between spectral data (structural correspondence), biological information (biological activity, taxonomy, chemosystematics, etc.) and geographical aspects (distribution and occurrence in Brazilian territory).

#### Technical validation for data quality

To ensure the quality of the data in the NuBBE_DB_, we used a method that encompasses several layers of validation. First, in collaboration with the CNPq, we conducted the aforementioned search of scientific papers to reduce bias and improve coverage. Second, to reduce errors, data are inserted via a web-based system with organized fields and drop-down menus. This platform allows simultaneous data entry by multiple remote users. The original papers are manually evaluated in order to extract information. All data entries are checked twice for integrity, and periodically, a re-evaluation is performed as part of our collaboration with the Royal Society of Chemistry ChemSpider Database^28^.

### Current status of compounds catalogued from Brazilian biodiversity

#### Natural product sources in Brazil

For the first time in Brazil, we can estimate the distribution of natural products within the Brazilian biomes, the relationship of this distribution with species occurrence and the association of biological activities with effects that govern Brazilian biodiversity (species, location, metabolic classes, etc.).

**Figure 2:**
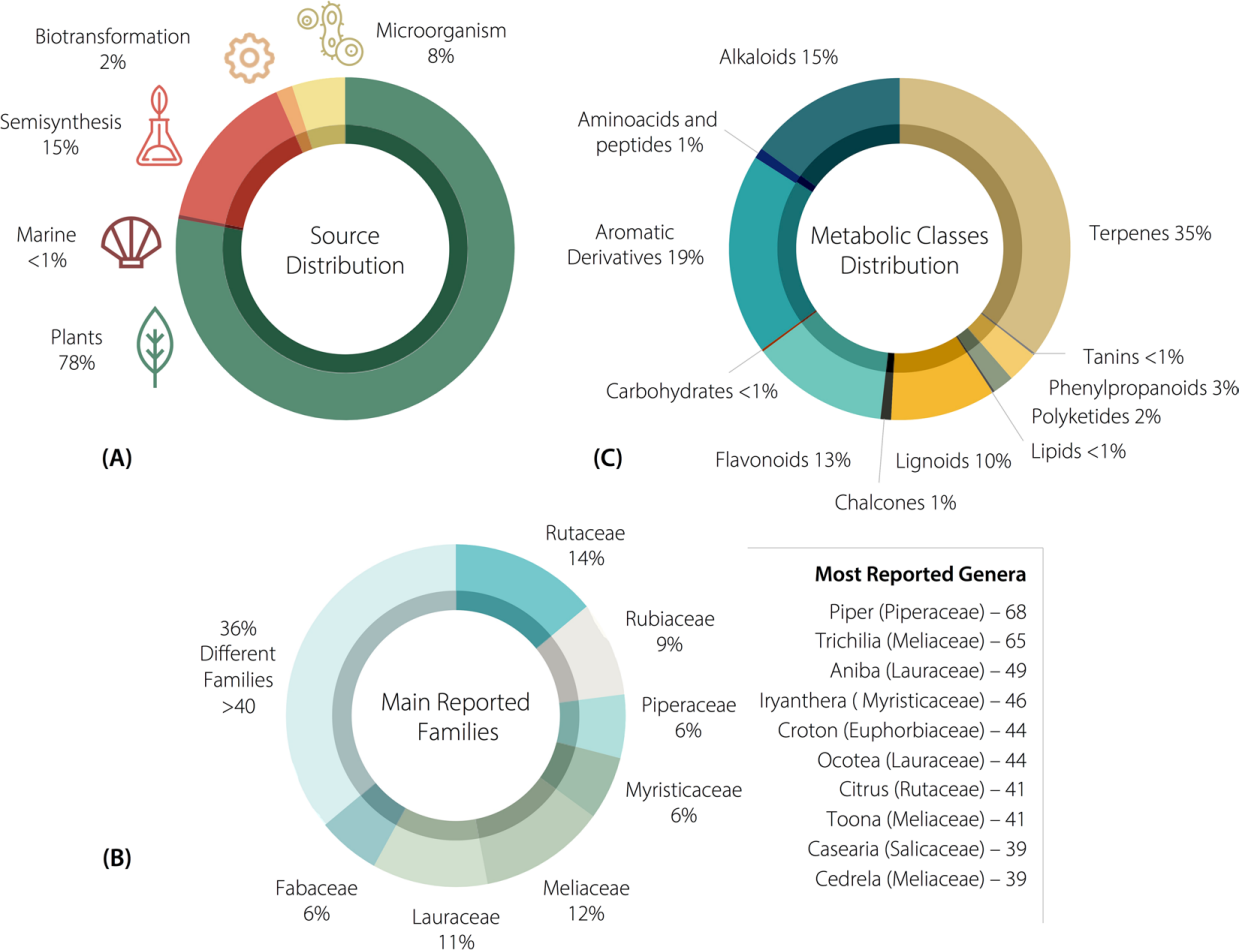
Statistics for the natural compounds from Brazilian biodiversity available in the NuBBE_DB_. (**A**) Sources of compounds. (**B**) The families from which the most compounds were identified, and the ten most prevalent genera. (**C**) Distribution of compounds by metabolic class.

Of the 2147 compounds (2147) in the NuBBE_DB_, 1688 were isolated from plants (78%), 325 are semi-synthetic products (15%), 109 were obtained from microorganisms (5%), 34 are biotransformation products (1.6%), and 8 are from the marine environment (0.2%). The corresponding data are shown in Fig. 2A.

Plants remain the main source of the studied natural products. However, since our last publication^37^, a remarkable growth of approximately 100% in the number of semi-synthetic products was observed. This result reveals the historical dynamics of natural product chemistry in Brazil. Most studies involving natural products that were conducted from the 1950s to the 1970s were led by Otto R. Gottlieb, Benjamin Gilbert and Walter Mors and focused on chemosystematics and bioprospecting from medicinal herbs and vascular plants (angiosperms)^48, 49^. Brazilian terrestrial microorganisms, such as plant endophytes, fungi, bacteria, plant rhizosphere microorganisms and marine organisms, were first studied in the 1980s and 1990s^50^. Despite the enormous biodiversity in Brazil, the lack of bioprospecting studies involving the other phylogenic kingdoms, i.e., Animalia, Archaea, Bacteria, Protozoa and Fungi, is evident.

#### Geographical distribution of compounds in Brazil

The incorporation of biogeochemical information about Brazilian species has positioned the NuBBE_DB_ as a unique chemical library. Although the Dictionary of Natural Products^51^ (DNP), Super Natural II^52^, AntiBase and MarinLit^53^ are robust and notable for natural product dereplication purposes, they do not describe the primary factors correlating species with biological activities and geographical disposition. The lack of this cross-referenced information hampers understanding of how environmental aspects (geography, climate and ecosystem) affect the metabolic profile of a given species, genus or family. The cross-referenced information regarding the taxonomy, geographical location and molecular descriptors of metabolites provided by the NuBBE_DB_ is beneficial in varied and important research areas, such as phenology, chemosystematics, ethnopharmacology and metabolomics.

**Figure 3:**
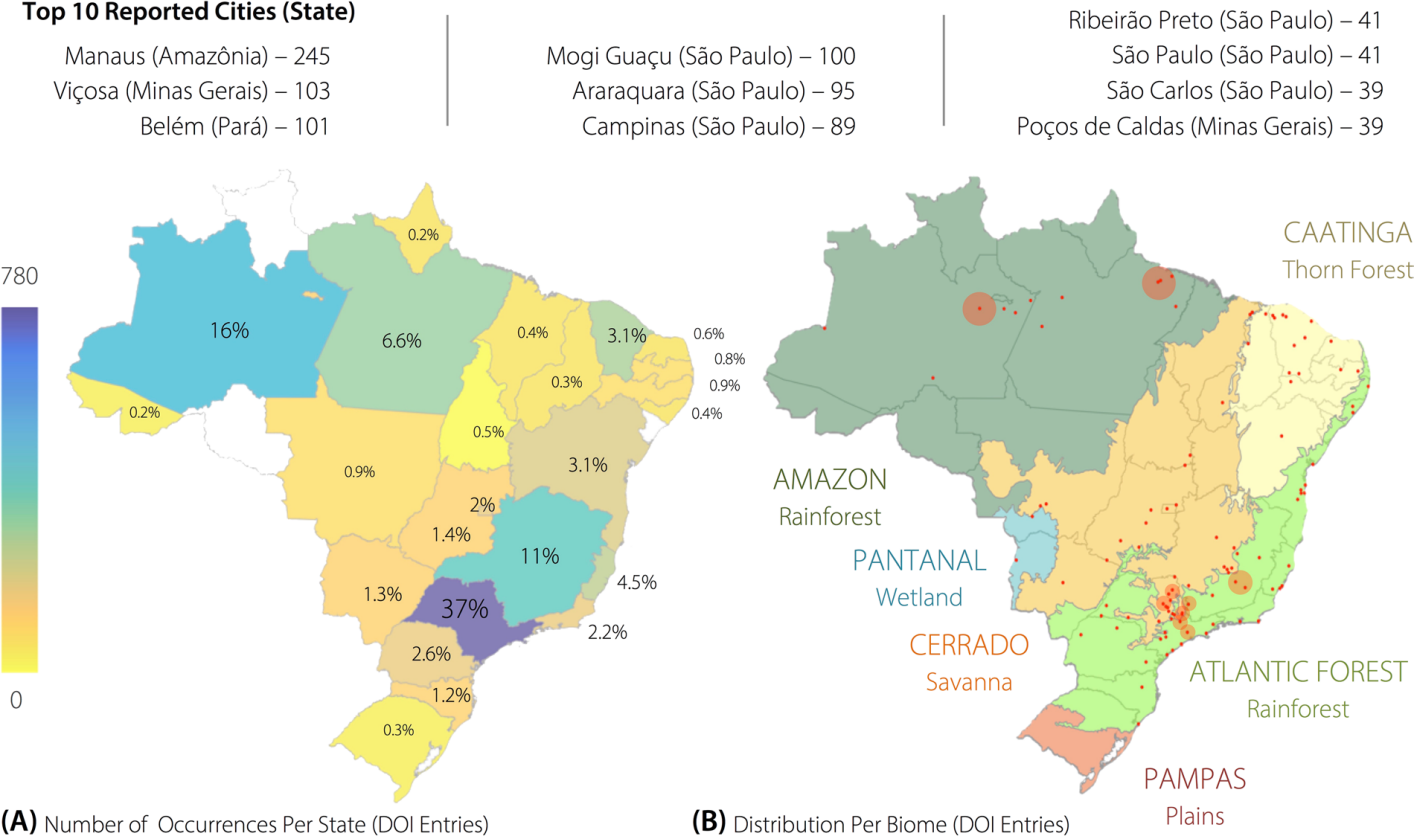
Representation of the distribution of NuBBE_DB_ compounds in Brazilian territory and its biomes. (**A**) Distribution of the species from which compounds in the NuBBE_DB_ were identified for each Brazilian State. (**B**) Species from which the compounds in the NuBBE_DB_ were identified are from all six different Brazilian biomes (the red dots represent the cities where these species were collected). The ten most frequently reported cities (upper part) are denoted in (**B**) as a red shadow (the size corresponds to the number of occurrences). Software used to create the map: Matlab R2016b (Mathworks, MA, USA).

The distribution of the geographic locations of the species from where the compounds in the NuBBE_DB_ were identified is illustrated in Fig. 3(A). Currently, compounds identified in species from almost all Brazilian states are available in the database (States: Acre – 0.2%, Alagoas – 0.4%, Amapá – 0.2%, Amazonas – 16%, Bahia – 3.1%, Ceará – 5.4%, Federal District – 2%, Espírito Santo – 4.5%, Goiás – 1.4%, Maranhão – 0.4%, Minas Gerais – 11%, Mato Grosso – 0.9%, Mato Grosso do Sul – 1.3%, Pará – 6.6%, Paraíba – 0.8%, Pernambuco – 0.9%, Piauí – 0.5%, Paraná, 2.6%, Rio de Janeiro – 2.2%, Rio Grande do Norte – 0.6%, Rio Grande do Sul – 0.3%, Santa Catarina – 1.2%, São Paulo – 36.9% and Tocantins – 0.1%); the exceptions are the states of Roraima, Rondônia and Sergipe.

The NuBBE_DB_ comprises compounds identified in species from all six Brazilian biomes. The geographic locations of the species are expressed with city and state descriptors. The top ten cities that appear in the NuBBE_DB_ are depicted in Fig. 3B. The Brazilian tropical savanna, called the Cerrado, and a portion of the Atlantic Forest characterize the vegetable physiognomy of the most commonly reported Brazilian state, São Paulo (Fig. 3A and B). Nevertheless, the last century has observed a dramatic loss in the biodiversity of this state, mainly because of the intensification of the urbanization process and the extensive development of monocultures, especially sugarcane. Thus, São Paulo became prevalent in the study of biodiversity not because of its nature but because of the state’s political policies and major investments in research through their well-managed science and technology-funding agency, FAPESP. Although Amazon is the second most studied state, most of the research was performed in universities located in the southeast region in Brazil (São Paulo, Rio de Janeiro, Minas Gerais and Paraná states). A comparison study shows that FAPESP invested approximately US$400 million in scholarships and research support in São Paulo State in 2015, while the MCTIC in Brazil distributed approximately US$2.6 billion among 26 states and the federal district. This total is equivalent to US$100 million per state, which is a quarter of FAPESP’s investment^54^.

#### Metabolic classes and taxonomic distribution

According to the taxonomic distribution, Rosidae followed by Magnoliidae and Asteridae are the most studied orders in the NuBBE_DB_ (Fig. 2B). The Rutaceae family represents 14% of all metabolites, followed by Meliaceae at 12%, Lauraceae at 11%, Rubiaceae at 9%, Fabaceae at 6%, Piperaceae at 6%, and Myristicaceae at 6%. The remaining 36% were distributed among more than 40 different families (Fig. 2B).

**Figure 4:**
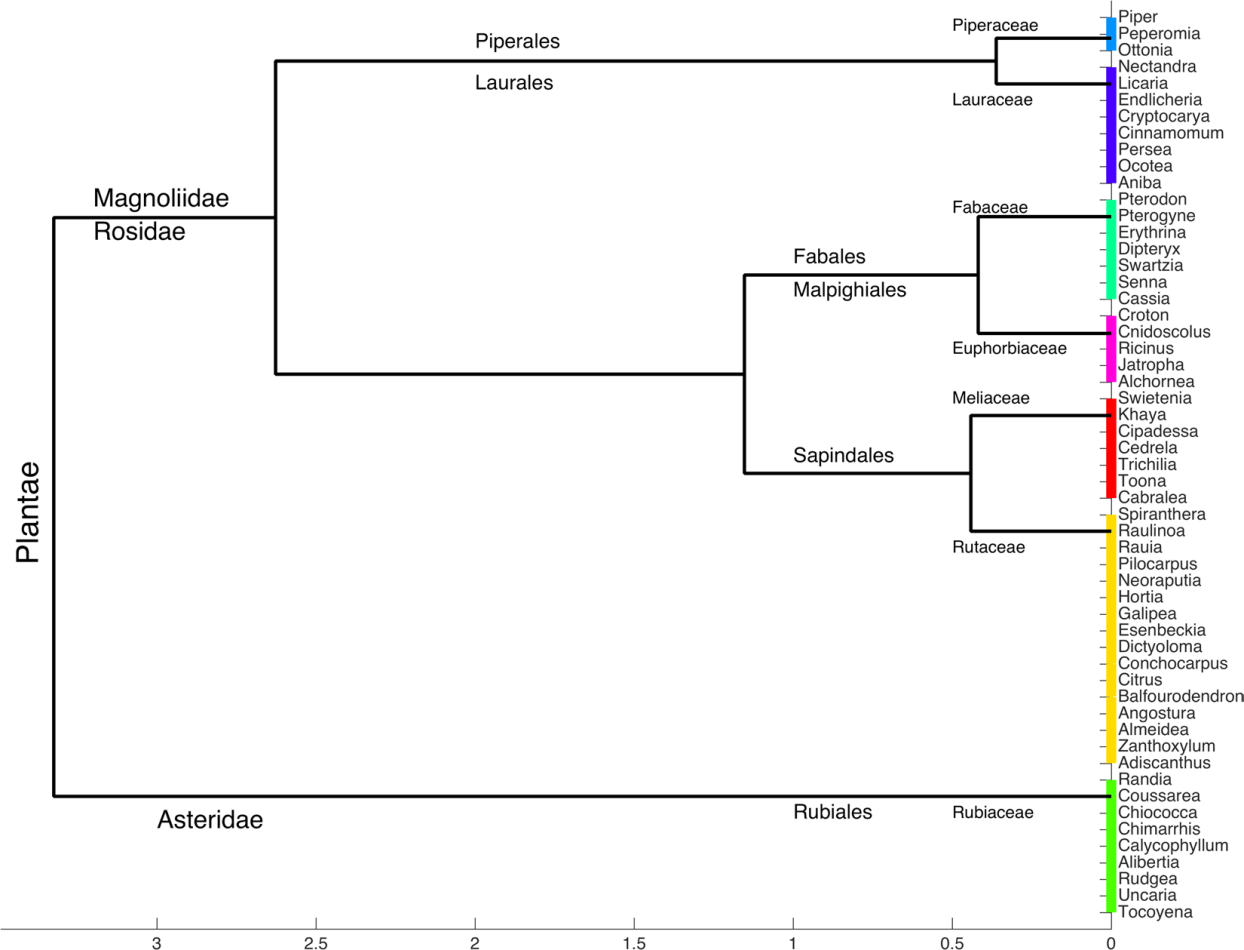
Hierarchical dendrogram representing the taxonomic structure (classes, orders, families and genera) of the most frequently reported species from which the compounds in the NuBBE_DB_ were identified.

*Piper* (Piperaceae) is the most populated genus, accounting for 68 entries, followed by *Trichilia* (Meliaceae) with 65 and *Aniba* (Lauraceae) with 49 (Fig. 2B). Currently, Rutaceae is the family with the highest number of reported genera (16 in total), followed by Rubiaceae and Lauraceae, with 9 and 8 genera, respectively (Fig. 4).

The most reported families in the NuBBE_DB_ are also registered in the GBIF as follows: Piperaceae - 180,276 registers, Rutaceae – 252,112, Myristicaceae – 44,798, Meliaceae – 117,687 and Lauraceae – 314,753. These families are mainly concentrated in pantropical regions, primarily in Central and South America, but some are also found in Oceania and South Asia^55^. These findings confirm the importance of this database for cataloguing endemic species from pantropical continents and their unique molecular and biological properties.

These results are also consistent with the distribution and occurrence of plant families found in Brazilian territory that are described in SiBBr (Brazilian Biodiversity Information System)^56, 57^. In SiBBr, Fabaceae, Asteraceae, Rubiaceae, Poaceae, Myrtaceae, Melastomataceae and Euphorbiaceae are the most frequently reported families in Brazil.

Classifying secondary metabolites is another important aspect of assessing the chemical diversity in species and biomes and estimating abiotic and biotic effects on metabolic production. The classification process in the NuBBE_DB_ was recently standardized according to Dewick’s biosynthesis theory^42^. Despite being created for plant biosynthesis, this classification also corresponds well to the other kingdoms.

**Figure 5:**
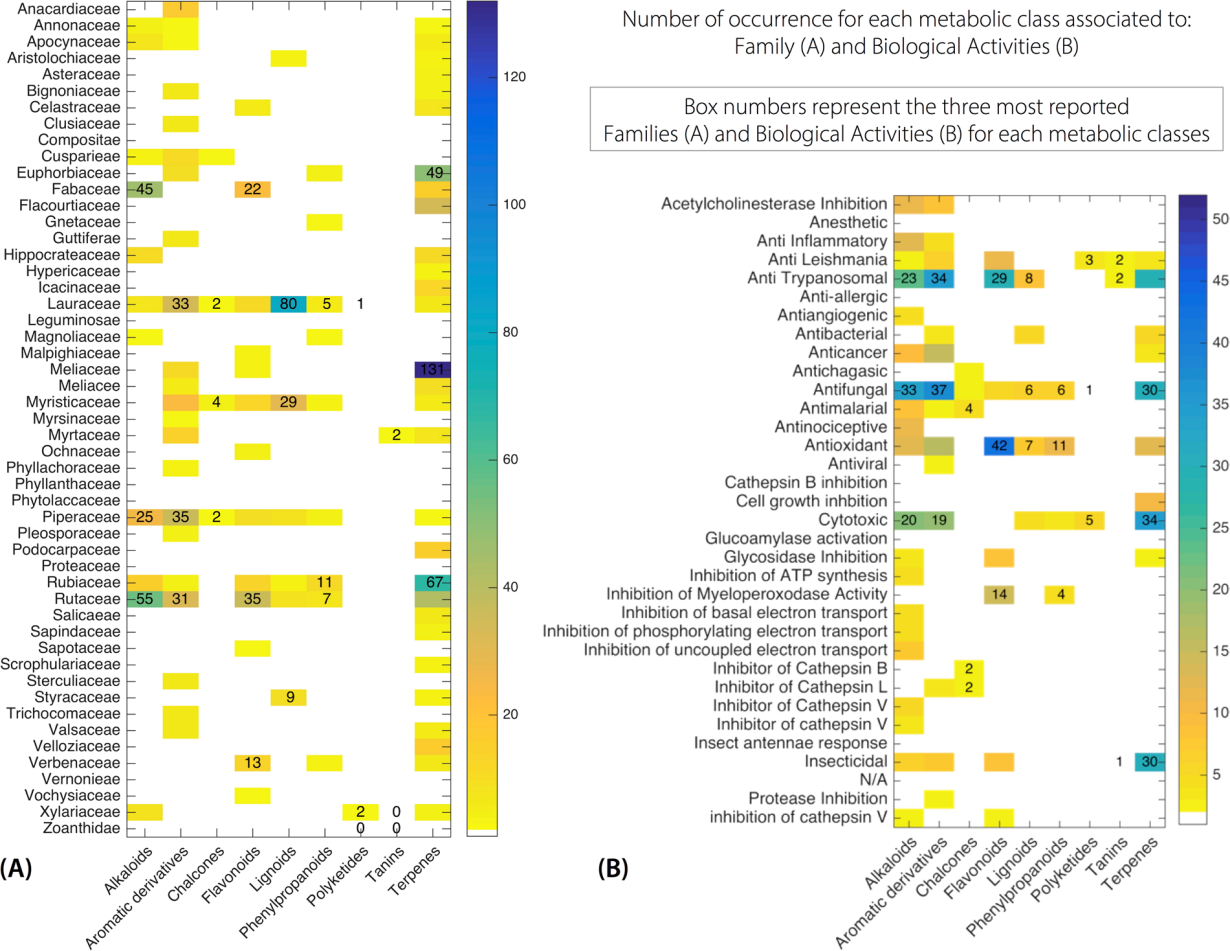
Heatmap of the occurrence of metabolic classes in (**A**) taxonomic groups (families) and (**B**) biological properties.

On the NuBBE_DB_ website, metabolic classes can be correlated with different information, such as species location, biological activity and taxonomy. Currently, 36% of all metabolites are classified as terpenes (identified mainly in the Meliaceae, Rubiaceae and Rutaceae families), 19% are associated with aromatic derivatives (encountered in Rutaceae, Piperaceae and Anacardiaceae), 15% are alkaloids (in Rutaceae, Fabaceae and Piperaceae), 13% are flavonoids (in Rutaceae, Lauraceae and Myristicaceae), 10% are lignoids (Lauraceae, Myristicaceae and Rutaceae), and the remaining 7% are distributed among 6 different classes (amino acids and peptides, chalcones, lipids, phenylpropanoids, polyketides and tannins) (Figs 2C and 5A).

The metabolic classes of the compounds from the NuBBE_DB_ can be correlated with family occurrence, allowing preliminary chemosystematics studies and assisting research associated with specific metabolic classes. For example, the main components of Fabaceae in the NUBBE_DB_ are alkaloids, flavonoids and terpenes (Fig. 5A). These metabolic classes are in agreement with the chemotaxonomic classification of the Fabaceae family reported by Wink and Waterman (1999)^58^. Amino acid derivatives, such as canavanine and lathyrane, and isoflavonoids are the most common biomarkers of Fabaceae^58^.

According to Wink and Waterman (1999), some coumarins and quinoline alkaloids are also taxonomic markers for the Rutaceae family^58^. These two groups are extensively represented by alkaloids and aromatic derivatives in the NuBBE_DB_ (Fig. 5A). The many Lauraceae-derived neolignans and arylpyrones present in the NuBBE_DB_ also denotes their chemotaxonomic value for the Lauraceae family^48^ (Fig. 5A). The correlation graphs shown in Fig. 5A demonstrate the potential of how natural products can assist in taxonomic studies.

#### Bioactivity and its relation to natural product occurrence

The database can be used to promptly identify relevant plants or metabolic classes associated with therapeutic effects. Among all metabolic classes, alkaloids comprise the highest number of bioactive compounds and are mainly associated with antifungal (33), antitrypanosomal (23) and cytotoxic (20) activities. Aromatic derivatives are primarily associated with antifungal (37), antitrypanosomal (34) and cytotoxic (19) activities as well. For flavonoids, the most common biological activities are antioxidant (42), antitrypanosomal (29) and myeloperoxidase inhibitory (14) activities. Phenylpropanoids have a similar profile: 11 have antioxidant activity, 6 have antifungal activity and 4 have myeloperoxidase inhibitory activity. For terpenes, cytotoxic (34), insecticidal (30) and antifungal (30) activities are the three most reported biological activities (Fig. 5B).

**Figure 6:**
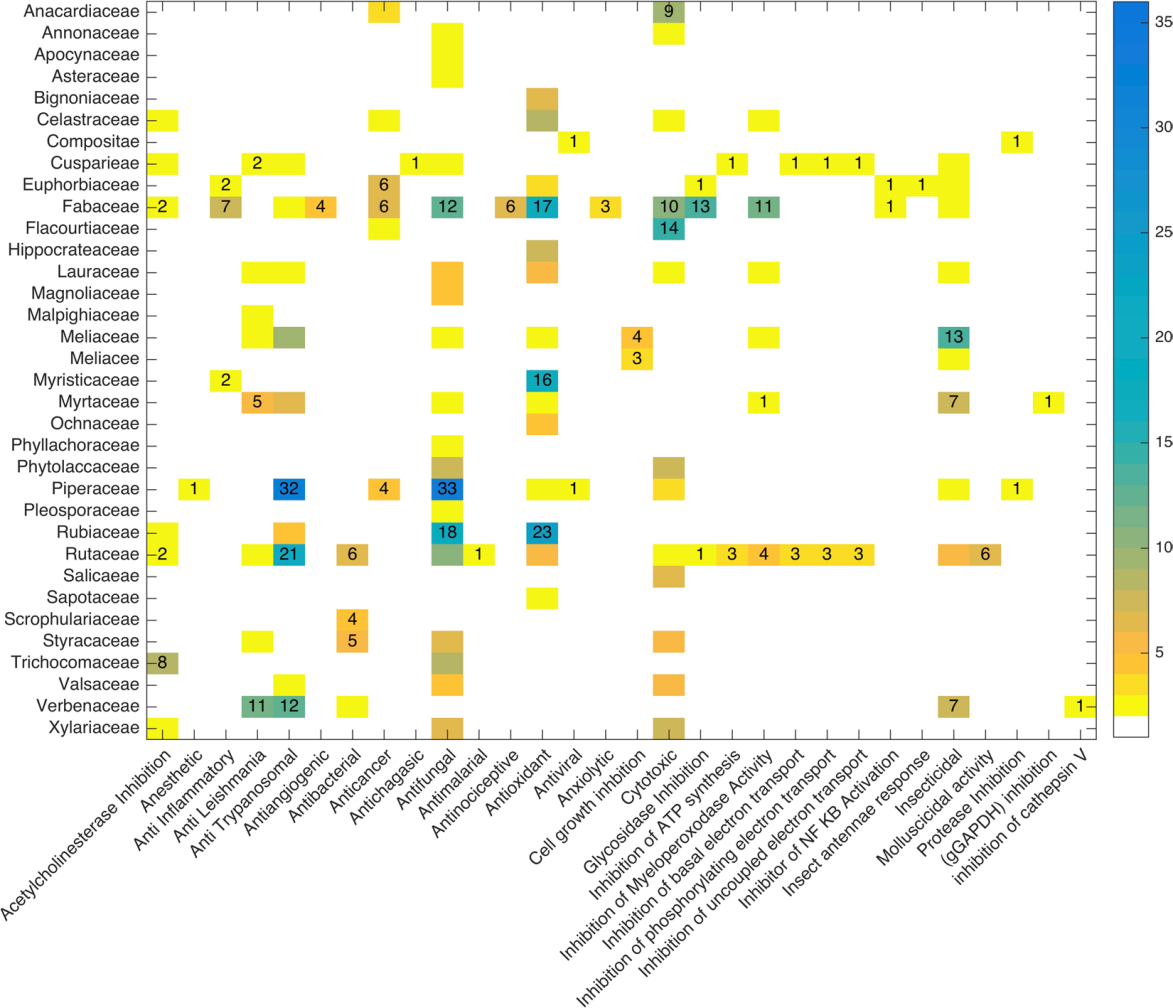
Heatmap of the relationships of compound biological properties with taxonomic groups (families).

On the website, it is possible to perform a filtered search associating biological activity and metabolic classes. For example, antitrypanosomal biological activity was searched and classified according to the number of occurrences in different metabolic classes. Aromatic derivatives (34) are the most reported, followed by alkaloids (23), flavonoids (29) and terpenes (28) (Fig. 5B). In addition, the families or genera related to a given biological activity can be determined. Among the 48 families, Rutaceae and Piperaceae have the most reports of antitrypanosomal and antifungal activities, Meliaceae is mainly associated with insecticidal activity, and Rubiaceae has primarily antifungal and antioxidant activities. Numerous biological activities, such as anti-inflammatory, antibacterial, antifungal, antioxidant, cytotoxic, glycosidase inhibitory and myeloperoxidase inhibitory activities, are displayed by compounds from Fabaceae (Fig. 6). However, these data are limited since only approximately 5% of the total chemical information on Brazilian biodiversity is available in the NuBBE_DB_. Additionally, only approximately 40% of these compounds have a reported bioactivity.

#### Drug discovery and medicinal chemistry descriptors

Brazilian plants, microorganisms and marine invertebrates are prominent sources of molecules and metabolic processes with scientific and socioeconomic value and have the potential, by means of pharmaceutical products, to contribute to an improved quality of life.

Molecular mass, number of hydrogen bond donors and acceptors, cLogP, nRotb, TPSA, and number of violations of Lipinski’s Ro5 are all useful descriptors for predicting the “drug-likeness” of small molecules and assisting with the first steps of bioavailability studies^59–62^. In our previous report, we explored the molecular features available in the NuBBE_DB_.

**Figure 7:**
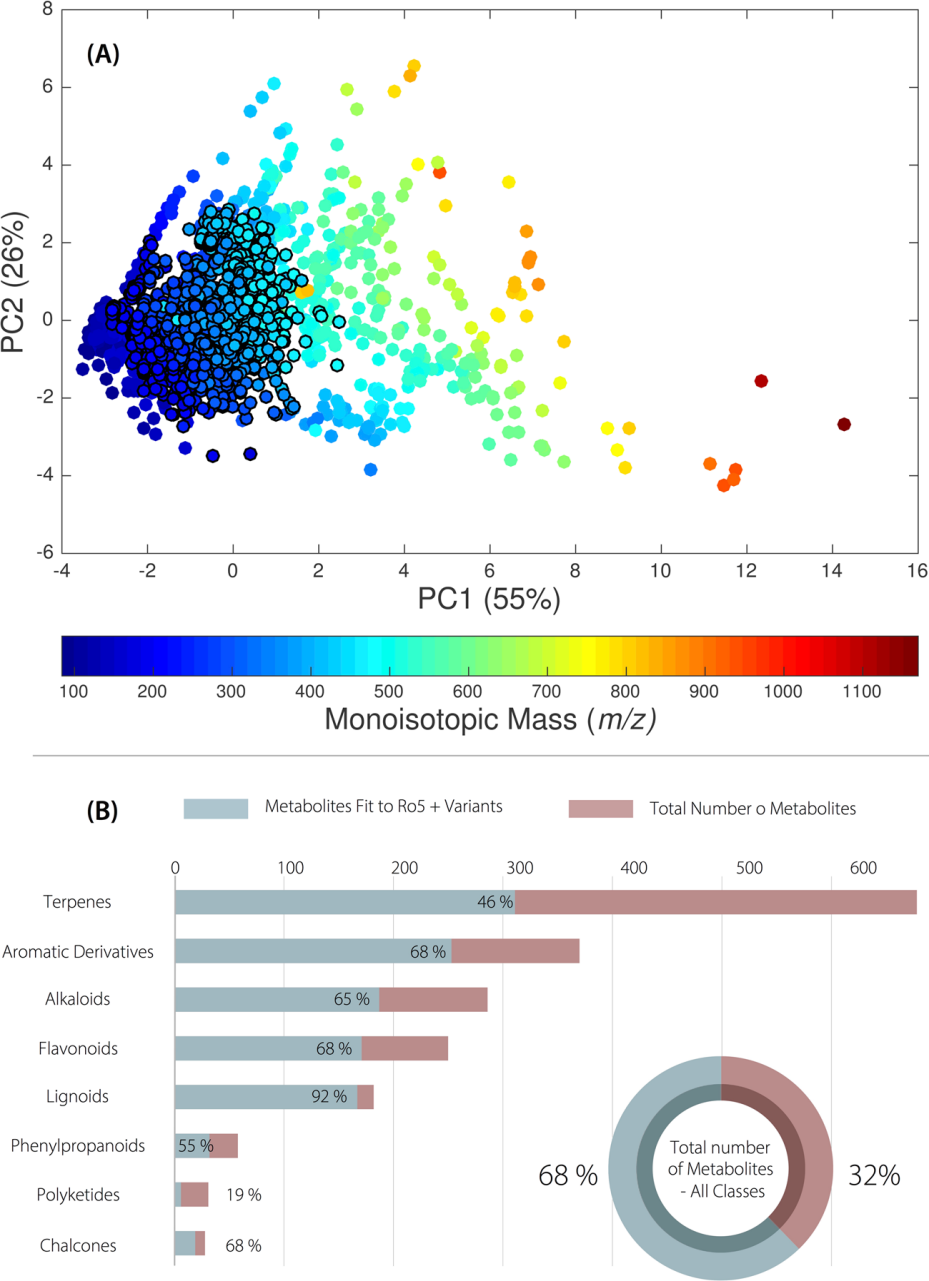
(**A**) Principal component analysis applied to molecular descriptors (physicochemical properties) of the compounds present in the NuBBE_DB_. The gradient of colors represents the compound distribution as a function of the monoisotopic mass variable. Black borderlines were added to denote compounds obeying the Lipinski-Veber rules. (**B**) Pie chart representing the total number of compounds (from all metabolic classes) that obey the Ro5 and Variant rules and bar graphs representing the compound distributions for different metabolic classes are shown.

In this update, we re-evaluate the chemical space and “drug-likeness” of the metabolites using Lipinski-Veber filters (Ro5 + Variants) in combination with chemometric tools, such as principal component analysis (PCA) (Fig. 7A). We performed PCA to evaluate the chemical properties that best characterize the chemistry of Brazilian natural products. For the first component (PC1), molecular mass and volume as well as TPSA can be used to assign a size-dependent relationship. For PC2, cLogP, number of hydrogen bond donors and acceptors, and nRotb express contributions from intermolecular forces. According to Veber^61^, reduced molecular flexibility and low polar surface area are important predictors of good oral bioavailability. Indeed, the PCA components support Veber’s statement with regard to the molecular distribution of chemical bonds and molecular arrangement. The NuBBE_DB_ metabolites are concentrated in a region with high potential for oral bioavailability.

Black borderlines denote molecules (dots) that obey the Lipinski-Veber rules (no more than 5 hydrogen bond donors, no more than 10 hydrogen bond acceptors, molecular weight from 180 to 500 Da, cLogP between −0.4 and + 5.6, less than or equal to 10 rotatable bonds and polar surface area no greater than 140 Ǻ^2^) (Fig. 7A). Approximately 1200 molecules fit in these filters, accounting for approximately 60% of all compounds in the database (Fig. 7A and B).

We also estimated which metabolic classes best fit the Lipinski-Veber rules (Ro5 + Variants) (Fig. 7B). Despite alkaloids being the most biologically active class in this database, lignoids are the most promising class regarding molecular bioavailability (92% of metabolites fit to Ro5 + Variants). These results demonstrate that the NuBBE_DB_ is not only a molecular repository but also a preliminary tool for drug discovery studies.

## Discussion

For the NuBBE_DB_, the last four years have seen substantial advances mainly associated with content expansion (increase of ca. 200%), source diversification, computational structure and NMR prediction tools. The database structure was also improved such that data are inserted in a homogeneous and standardized manner, reducing errors. We consider data quality to be an important factor in the NuBBE_DB_. Therefore, all information is checked twice, and collaborations have been established (e.g., with ChemSpider) to certify the content. Compared with software packages, the NuBBE_DB_ has several advantages, including not requiring installation, allowing data exploration using multiple rational alternatives and being designed to assist scientists with different expertise.

The NuBBE_DB_ has significantly contributed to the mapping of Brazilian chemical biodiversity. Currently, more than 2000 compounds with taxonomic information, geographical location and species information are catalogued. Because of the extensive studies on vascular plants performed between the 1950s and 1970s, plants are the most common source of compounds in the database. The states of São Paulo and Amazon have the highest number of studied species, and consequently, the Cerrado (tropical savanna) and the Amazon Rain Forest are the most studied biomes. GBIF and SiBBr reports are in accordance with the natural product statistics from the NuBBE_DB_ and reinforce the unique importance of this repository as a source of compounds from endemic Brazilian pantropical families, such as Piperaceae, Rutaceae, Myristicaceae, Meliaceae and Lauraceae.

The connection of different topics involving biological properties, taxonomy and metabolic classes is a unique feature of the NuBBE_DB_ and enables a general view of the relationship between metabolic distribution and geographic physiognomy, as well as multifactorial events, such as climatic and ecological changes. In this preliminary study, Fabaceae was found to be the family with the highest number of biological properties, primarily because of the alkaloid metabolites found in this family. The *Piper* genus had a remarkable propensity for antifungal and antitrypanosomal activities, which were also associated with nitrogen-containing compounds. Another interesting aspect is the oral bioavailability properties demonstrated by lignoids.

Notably, the NuBBE_DB_ has not only provided an evaluation of Brazilian chemical biodiversity but also revealed the limited number of chemical and bioprospecting studies involving natural sources other than plants. Marine natural products have emerged in the last decade as a promising natural product source for anticancer agents such as cytarabine and trabectedin. Despite Brazil being the nation with the 16^th^ longest maritime coast in the world (approx. 7.5 thousand kilometers), much of its seas remain underexplored. Terrestrial biomes such as Caatinga, Pampas and Pantanal are also not well investigated.

In the next few years, we expect that the NuBBE_DB_ will increase the extent and diversity of its content and that robust predictions and modeling of Brazilian biodiversity will occur, thus assisting in the interpretation of the multifactorial events that rule Brazilian ecosystems. We believe that this database will serve as a useful “knowledge base” for drug discovery, metabolomics and plant science projects through its ability to connect chemistry, biology and informatics. We also expect that the database will serve as an information source for conservation policies and the technological and socioeconomic development of communities that use Brazilian biodiversity products.

## Methods

### NuBBE database website structure

The NuBBE Web system is installed on a Linux server with Apache Tomcat as the Web server and PostgreSQL as the relational database server. The Web interface is implemented using standard Web technologies such as HTML, CSS and JavaScript (AJAX), while the server itself is implemented using Java/Servlets with Hibernate, an object-relational mapping database framework. The data set is stored in the PostgreSQL database, including text-based, graphics and spectral files. The molecular drawing interface is provided by WebME/Molinspiration^63^, and the substructure search engine is provided by Chemistry Development Kit (CDK)^37, 64^.

The 2D image structure, IUPAC name and monoisotopic mass were generated using the Marvin package from ChemAxon^65^. Molecular features and physicochemical parameters were predicted using *mib* batch molecule processing, available as part of the web-based Molinspiration software^63^. This predicted information also includes molecular mass, molecular volume, numbers of hydrogen bond donors and acceptors, octanol/water partition coefficient (cLogP), number of rotatable bonds (nRotb), topological polar surface area (TPSA) and number of violations of Lipinski’s rule of 5 (Ro5). The 3D chemical structures (.MOL2 and .MOL format files) and InChI and InChIKey codes are generated using Open Babel^66^. In addition, the simulated ^1^H and ^13^C NMR spectra were generated by *H and C NMR predictors command line* provided by Advanced Chemistry Development, Inc. (ACD/Labs, Canada).

### Data Analysis

The distribution graph of compounds by source (i.e., plants, microorganisms, marine organisms and biotransformation and semisynthetic products) presented in Fig. 2 was generated by calculating the ratio between the number of compounds from each source and the total sum of compounds. The distribution graphs of metabolic classes and families were similarly generated. The cities, states and maps for all geographic shapefiles (.shp) were obtained from the Brazilian Institute of Geography and Statistics (IBGE) website^67^.

A map of the metabolite distributions in Brazilian states and cities (Fig. 3) was generated by calculating correlation functions between geographic coordinates and metadata obtained for each metabolite (source and locality) using graphical functions from Matlab Mapping Toolbox^68^. For the dendrogram shown in Fig. 4, the species were organized according to Cronquist’s^69^ plant taxonomy and arranged according to Euclidean distance and the Ward linkage grouping method. Heatmaps and principal component analysis (PCA) charts were created using cross-linked information (i.e., biological activity, source, species, and metabolic class) available on the website. For the heatmaps (Figs 5 and 6), the image function from Matlab R2015 and graphical features from Microsoft Power Point for Mac 2011 (v. 14.0.0) were used. For the PCA chart (Figure 7A), the data set (including the monoisotopic mass, cLogP, TPSA, Lipinski rule violations, H-bond acceptors and donors, rotational bonds and molecular volume) was normalized and auto-scaled using the Statistics toolbox from Matlab R2015.

### Data Availability

All data available in the database and used for the production of figures can be downloaded from http://nubbe.iq.unesp.br/portal/nubbedb.html.
